# Sleep facilitates long-term face adaptation

**DOI:** 10.1098/rspb.2013.1698

**Published:** 2013-10-22

**Authors:** Thomas Ditye, Amir Homayoun Javadi, Claus-Christian Carbon, Vincent Walsh

**Affiliations:** 1 Institute of Cognitive Neuroscience, University College London, London WC1N 3AR, UK; 2 Section of Systems Neuroscience, Department of Psychology, Dresden University of Technology, Dresden, Germany; 3 Department of General Psychology and Methodology, University of Bamberg, Bamberg, Germany

**Keywords:** adaptation, sleep, learning, faces, figural after-effects, plasticity

## Abstract

Adaptation is an automatic neural mechanism supporting the optimization of visual processing on the basis of previous experiences. While the short-term effects of adaptation on behaviour and physiology have been studied extensively, perceptual long-term changes associated with adaptation are still poorly understood. Here, we show that the integration of adaptation-dependent long-term shifts in neural function is facilitated by sleep. Perceptual shifts induced by adaptation to a distorted image of a famous person were larger in a group of participants who had slept (experiment 1) or merely napped for 90 min (experiment 2) during the interval between adaptation and test compared with controls who stayed awake. Participants' individual rapid eye movement sleep duration predicted the size of post-sleep behavioural adaptation effects. Our data suggest that sleep prevented decay of adaptation in a way that is qualitatively different from the effects of reduced visual interference known as ‘storage’. In the light of the well-established link between sleep and memory consolidation, our findings link the perceptual mechanisms of sensory adaptation—which are usually not considered to play a relevant role in mnemonic processes—with learning and memory, and at the same time reveal a new function of sleep in cognition.

## 1. Introduction

The integration of new experiences into our current representation of the world is a core function of perception. One mechanism that facilitates this process is adaptation, based on constant adjustments of the sensory systems following input. Neurons that are sensitive to a given stimulus show attenuated responses as a result of repeated exposure. This shift of a neuron's tuning function allows for differential processing of subsequent stimuli that are most likely in a given environment, facilitating a highly efficient system with maximal sensitivity [1,2]. Adaptation is usually studied in the form of negative figural after-effects, such as the motion after-effect [3], tilt after-effect [4] and face adaptation [5]. These effects share common characteristics [6], suggesting adaptation to be a generalized property of the nervous system that is fundamental to all perceptual analyses [7].

Although mainly studied at short timescales (i.e. milliseconds to seconds) adaptation effects can last for minutes [1,8], days [9] and even weeks [10–12]. Long- and short-term effects can co-occur [1,10,13,14]. For example, in the McCollough effect [15], the short-term component shows strong and fast build-up that decays quickly when adaptation has stopped, whereas the long-term component is weak, slow and infinite, showing no signs of decay or saturation [14]. Similar dissociations have been shown in contrast and chromatic adaptation [1,10], and have been suggested to operate also in face adaptation [6,11,16]. However, the underlying mechanisms of such long-term perceptual baseline shifts and the factors that contribute to their permanence are still largely unknown.

Here, we tested how long-term face adaptation is maintained in the visual system. Based on the well-established association of sleep and memory consolidation [17–19], we hypothesized that sleep may contribute to the integration of adaptation-induced long-term changes in neural function. Sleep plays a central role in skill acquisition and the formation of memories. The consolidation of both procedural [20] and declarative [21] information is facilitated by and partly dependent on sleep. Sleep can also be beneficial to other forms of learning such as insight [22], perceptual learning [23] and statistical learning [24].

Low-level after-effects (i.e. after-effects based on basic stimulus attributes such as orientation, colour and motion which are associated with early stages of the visual processing hierarchy) can persist longer when there is no intervening visual stimulation between adaptation and test—a phenomenon known as ‘storage’ [3,25–27]. Storage has not been shown in face adaptation and it is possible that higher-level after-effects (i.e. after-effects based on more complex stimuli such as scenes or faces) may be affected by sleep or intervening visual stimulation in ways that are different from those of the low-level effects tested before. Moreover, storage on the basis of reduced visual interference cannot account for the very long-lasting adaptation effects reviewed above. Such effects are more likely to result from active consolidation mechanisms that are common in learning and memory and were shown to be partly sleep-dependent.

**Figure 1.**
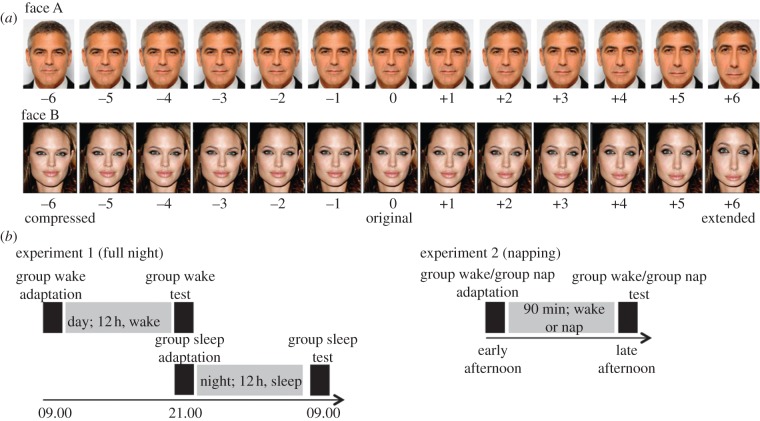
Stimuli and design. (*a*) The range of stimuli from highly compressed (*k* = −6) to highly extended (*k* = +6) created from a frontal view image (original) of George Clooney (face A) and Angelina Jolie (face B). (*b*) Design of the study for experiments 1 and 2. (Online version in colour.)

We studied the effects of sleep on face adaptation. During adaptation, participants viewed distorted images of famous actors (figure 1*a*). The most strongly extended images (*k* = +6) were viewed repeatedly for about 25 min. This adaptation procedure was followed by an interval that covered 12 h in experiment 1 (full night) and 90 min in experiment 2 (napping; figure 1*b*). In both experiments, one group of participants stayed awake during this interval and another group of participants slept. In experiment 2, participants of the wake group were blindfolded to prevent visual input. Finally, a testing procedure that required participants to judge the distortion levels of all pictures in the stimulus continuum was used to determine the size of after-effects induced by adaptation. Napping—usually in the form of a 60–120 min sleep period during the day—has been shown to affect skill acquisition and memory consolidation in a way similar to that of longer sleep periods [28].

## 2. Experimental procedures

### (a) Participants

Forty (experiment 1; 20 female; mean age: 23.2 years, ranging from 18 to 34 years) and 36 (experiment 2; 17 female; mean age: 22.4 years, ranging from 19 to 32 years) healthy university students participated in the study. All participants were right-handed, had normal or corrected-to-normal vision and were naive to the purpose of the experiment. Participants gave written informed consent, and the study was approved by the University College London (UCL) ethics committee.

### (b) Task and stimuli

Frontal view colour photographs of two famous United States American actors were selected; one picture per person, one male, one female: George Clooney (face A) and Angelina Jolie (face B). The original images (*k* = 0) were used to generate a continuum of configurally distorted images including six versions that were gradually compressed with respect to the original image (*k* = [−6 … − 1]) and six versions that were gradually extended (*k* = [+1 … + 6]; figure 1*a*). This manipulation was achieved by a nonlinear transformation of the original face (*k* = 0). Please see the electronic supplementary material for a description of the algorithm used for the generation of stimuli in Matlab (2010b, The MathWorks, Natick, MA). This method resulted in a set of 13 images per face that differed in their spatial configurations of facial features but were still recognizable as the same individual, as confirmed by pilot data (*n* = 4).

During adaptation, all participants were exposed to the strongly extended versions (*k* = +6) of either face A or face B (experiment 1; randomized across participants) or face A only (experiment 2). On each trial, the face was shown in a random location on a screen. Four presentation durations (0.5, 2, 4, 6 s) counterbalanced with three image sizes (pixels; small: 180 × 240; medium: 240 × 320; large: 300 × 400) were used to reduce predictability of the task and to control for retinal effects of size and position. Image presentation was preceded by a fixation cross on white background (1 s). The adaptation stimulus was presented, and the task was to press a button as quickly as possible as soon as the image disappeared. Four cycles of 12 trials were presented in randomized order in the course of one block of trials. There were six blocks with a break of 30 s between blocks. This procedure resulted in a total exposure time of 900 s.

At test, all 13 pictures of the distortion continuum (*k* = [−6 … + 6]) were presented in randomized order in the centre of the screen for a duration of 2 s each. After the presentation of each stimulus, participants were then asked to indicate whether they perceived the image shown as either compressed or extended (two alternative forced choice) by pressing the corresponding buttons on a keyboard. This randomized presentation of all 13 images was repeated for four (experiment 1) or 12 (experiment 2) times in one continuous block of trials. All statistics are based on the full set of available data of both experiments.

The experiment was controlled by Matlab (The MathWorks) using the Psychophysics Toolbox extensions [29,30] on a PC running Windows XP. Stimuli were presented on a 17 inch monitor with a screen resolution of 1024 × 768 pixels.

### (c) Study design and procedures

Experiment 1 comprised a full night design (figure 1*b*). One group of participants (*n* = 20; sleep group) was adapted at 21.00 and was tested at 09.00 in the morning on the following day. Participants of this group were asked to have a good night of sleep with approximately 8 h of sleep that night. A second group (*n* = 20; wake group) adapted at 09.00 and was tested at 21.00 in the evening of the same day. Participants of this group were instructed to have a ‘normal’ day without sleeping or napping at any point in between the two parts of the experiment.

In experiment 2, a napping procedure was used (figure 1*b*). Participants of all conditions were invited to the laboratory at 13.00 or 16.30 in the afternoon. Each session started with preparing the electroencephalogram (EEG) followed by the adaptation procedure. After this, one group of participants (*n* = 12; sleep group) took a nap of 90 min which is the average time taken to complete one full sleep cycle. Another group (*n* = 12; wake group) was blindfolded immediately after adaptation for a time period of 90 min. While being blindfolded, for entertainment purposes, participants of the wake group were listening to three 30 min episodes of the British Broadcasting Corporation Radio 4 ‘The infinite monkey cage’ science podcast series (http://www.bbc.co.uk/programmes/b00snr0w). EEG was monitored online to ensure participants remained awake. Following this interval of 90 min, participants of both groups completed the test phase of the experiment. All participants in experiment 2 were adapted to the same face. We used a conservative approach and selected the face that showed the numerically smallest effect in experiment 1: face A. An additional group of participants served as a control group (*n* = 12; immediate group). For this group, there was no interval between adaptation and test. Instead, the test phase followed immediately after the adaptation procedure.

### (d) Electrophysiological measures

EEG was recorded continuously from 64 active electrodes with a BioSemi active-two amplifier system (BioSemi, Amsterdam, The Netherlands) placed according to the 10–20 electrode placement system. To monitor eye movements and blinks, horizontal and vertical electrooculograms (EOG) were recorded. Electromyogram (EMG) signals were recorded using two active electrodes placed over the chin. Raw EEG, EOG and EMG were sampled at 512 Hz with 12-bit resolution. Two electrodes were used as reference and ground electrodes. An additional electrode on the tip of the nose was used as reference.

### (e) Statistical analysis

#### (i) Behavioural data

In both experiments, sleepiness ratings (Stanford sleepiness scale) were subjected to a 2 × 2 mixed-design analysis of variance (ANOVA) with session (adaptation/test) as within subject and group (sleep/wake) as between subject independent factors. An independent sample *t*-test was run to compare average group response times during adaptation. The dependent variable in the analysis of individual behavioural adaptation effects was participants' fitted mean evaluations of the original face (*k* = 0) at test (fitted mean rating, FMR). FMRs were achieved by fitting logistic psychometric functions, 1 ∼ (1 + exp(−**β**(*x* − **α**))) with **α** indicating the point of ambiguity and **β** indicating the slope of the curve (Palamedes toolbox for Matlab [31]).

In both experiments, the FMRs at *k* = 0 of both sleep and wake groups were first tested against the theoretical point of ambiguity at *k* = 0 to assess generic effects of adaptation for each group separately. This criterion corresponded to the point of maximal ambiguity (no perceived compression or extension) which was the expected average percept of the original face prior to adaptation. One-sample *t*-tests were calculated for each group using one-tailed, uncorrected testing, given our *a priori* hypothesis about the direction of the effects. To test for group differences in the size of the adaptation effects a 2 (group: sleep/wake) × 2 (adaptation face: face A/face B) × 2 (gender of the participant: male/female), univariate ANOVA was calculated for experiment 1. In experiment 2, a 3 (group: nap/wake/immediate) × 2 (gender: male/female) ANOVA was calculated. In experiment 2, there was no factor adaptation face, because all participants adapted to face A.

#### (ii) Sleep data

Pre-processing of sleep data included downsampling to 256 Hz, and bandpass filtering of the EEG (0.3–35 Hz), EOG (0.1–35 Hz) and EMG (40–120 Hz) data using EEGLab toolbox in Matlab [32]. The data were then scored manually by two researchers independently in 30 s epochs according to the sleep scoring standards by Rechtschaffen & Kales [33] using the SleepSMG toolbox (Walker laboratory; http://sleepsmg.sourceforge.net/) running in Matlab. Concordance rating of the two examiners was 83.4%. The researchers then re-examined all epochs that were rated inconsistently and agreed on a consistent rating for each epoch. The total number of epochs from each sleep stage was used as an absolute as well as relative (compared with total sleep time) measure of the duration of each sleep stage which was then correlated with measures of individual behaviour using bivariate Pearson correlations (uncorrected).

#### (iii) Spindle analysis

An automatic procedure similar to other studies [34] was used to detect slow and fast spindles. Three representative channels Fz, Cz and Pz were selected for the analysis. It has been shown that spindles activity is most pronounced on these channels [35]. Only stages 2–4 were included in the analysis. Data were filtered with a bandpass filter 9–12 Hz for slow and 12–15 Hz for fast spindles. Subsequently, a thresholding algorithm was used to detect the spindles [35]. The root mean square (r.m.s.) of the filtered signal was calculated using a moving window of 0.2 s. The threshold for spindle detection in the r.m.s. signal was set to 1.5–2.5 standard deviations (s.d.) of the filtered signal. Spindles shorter than 0.5 s and longer than 3 s were excluded from further analysis.

#### (iv) Frequency analysis

Data were filtered using a 0.15–48 Hz bandpass filter. Three representative channels Fz, Cz and Pz were selected for this analysis. Movement and awake epochs were excluded from the analysis. Each epoch was analysed separately using four overlapping windows of 10 s. Fast Fourier transform (FFT) was calculated for each window in each epoch and each channel. Subsequently, the absolutes of the FFT of all the windows in all the epochs were averaged over each channel. The logarithm of this mean in each frequency bin was correlated with FMRs.

## 3. Results

### (a) Experiment 1: full night

All participants were familiar with the adaptation faces. All participants of the sleep group (*n* = 20) had a night of approximately 8 h of sleep during the interval between adaptation and test, based on self-reports. Participants of the wake group (*n* = 20) did not sleep or nap at any time during the interval, based on self-reports. Response times during adaptation were comparable for both groups (independent sample *t*-test; *p* = 0.976; n.s.). A 2 (group: sleep/wake) × 2 (session: adaptation/test) mixed-design ANOVA was used to analyse participants' ratings of their subjective sleepiness. There were no significant main effects or interactions (all *p* > 0.05) indicating similar levels of sleepiness in both groups and sessions.

To determine the size of the adaptation effect, logistic psychometric functions were fitted on participants responses. Subsequently, participants' evaluations of the original face (*k* = 0) at test were used as a measure of adaptation (FMR). One participant out of 40 was excluded from all analyses on the basis of individual responses that deviated more than 2.5 s.d. from group average (sleep group; face B). Under conditions without adaptation, the original familiar face should be perceived as neither compressed nor extended therefore arriving at an average rating of 0.5 on a scale from 0 to 1. Based on previous literature [5], we expected to see a shift in participants' evaluation of the original face towards compressed after adaptation to extended faces (*k* = +6), i.e. the direction opposite to the adapting stimulus. FMR were *M* (s.d.) = 0.20 (0.22) in the sleep group and 0.39 (0.22) in the wake group, both significantly different from the hypothetical origin of 0.5 (one-tailed one-sample *t*-tests: *t*_18_ = 6.18; *p* < 0.001; Cohen's *d* = 1.37 and *t*_19_ = 2.31; *p* = 0.016; *d* = 0.52, respectively). Because our assumption of an average FMR of 50% has not been empirically tested, the result of the wake group, which falls within a reasonable proximity of this critical value has to be treated with care and will not be further interpreted. Taken together, these findings indicated an adaptation effect that was measured 12 h after adaptation in, at least, the sleep group, replicating previous reports of long-term effects in face adaptation [11].

**Figure 2.**
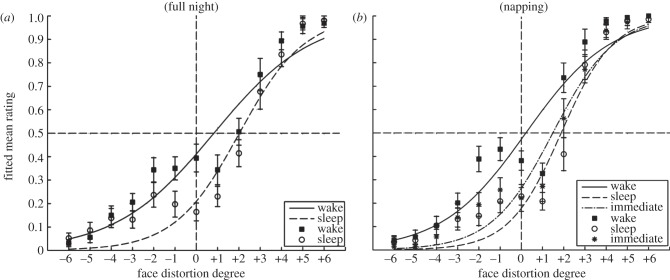
Behavioural results. Participants’ mean ratings in (*a*) experiment 1 and (*b*) experiment 2. Psychometric functions were fitted to the mean ratings. Error bars indicate 1 s.e.m.

The size of the adaptation effect was significantly larger in the sleep group compared with the wake group, as indicated by a significant main effect for group in a 2 (group: sleep/wake) × 2 (gender of participant: male/female) × 2 (adaptation face: face A/face B) univariate ANOVA, *F*_1,31_ = 7.26; *p* = 0.011; 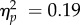 (figure 2*a*). This shows that sleep had a facilitating effect on the retention of the perceptual shifts induced by adaptation. Because each participant adapted to only one single face, we also tested for the effects of adaptation face (A and B) and gender. There were no significant main effects or interactions (all *p* > 0.05).

In summary, experiment 1 showed that participants who experienced a period of sleep after adaptation exhibited a stronger after-effect compared with those who stayed awake. However, in this experiment, participants of different groups were tested at different times of the day. In addition, participants from the wake group were more likely to encounter and interact with natural faces during the day than the participants from the sleep group during the night. These imbalances between groups provides an alternative explanation for the reported findings that is not dependent on sleep. Therefore, in experiment 2, a napping paradigm was used instead of full night sleep. The wake group participants' vision was completely blocked during the interval to control for interference effects (figure 1*a*).

### (b) Experiment 2: napping

Experiment 2 used a napping paradigm to keep the time of day for adaptation and test constant for all participants and to control for exposure to natural faces during the interval period.

Similar to experiment 1, all participants were familiar with the adaptation face. Sleep EEG data showed that all participants from the nap group slept for an average duration of 80 min, ranging from 61 to 89 min. Analysis of response times confirmed that there were no systematic differences between nap and wake groups during adaptation (independent sample *t*-test; *p* = 0.386; n.s.). In addition, sleepiness ratings were similar across groups as shown by a non-significant main effect of group (*p* > 0.05; n.s.) in a 2 (group: sleep/wake) × 2 (session: adaptation/test) mixed-design ANOVA.

We calculated a 3 (group: nap/wake/immediate) × 2 (gender: male/female) univariate ANOVA on participants' fitted responses to the original face at *k* = 0. There was a significant main effect of group, *F*_2,30_ = 3.32; *p* = 0.05; 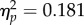, which was driven by an adaptation effect that was lager in the nap group (*M*(s.d.) = 0.20(0.19)) compared with the wake group (0.43(0.22); figure 2*b*), as confirmed by Bonferroni corrected post hoc comparisons (*p* = 0.047). There were no other significant main effects, interactions and pairwise comparisons in this analysis (all *p* > 0.05). Similar to experiment 1, one-sample *t*-tests versus the expected mean rating without adaptation (i.e. 0.5) showed that only the napping group showed significant adaptation towards compressed, *t*_11_ = 5.63; *p* < 0.001; *d* = 1.63 and *t*_11_ = 1.11; *p* = 0.145; n.s., respectively; one-tailed testing.

#### (i) Sleep data

**Figure 3.**
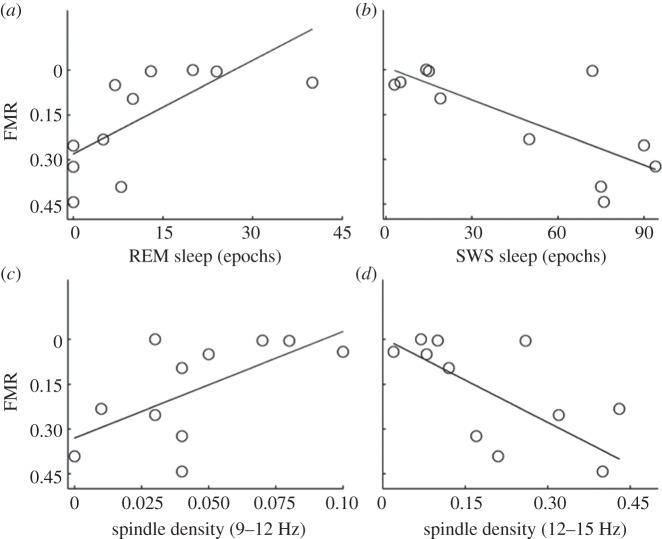
Sleep parameters. Post-sleep adaptation effects correlated significantly with sleep parameters: (*a*) REM sleep duration, (*b*) SWS duration, (*c*) density of slow spindles, and (*d*) density of fast spindles. Lower FMRs indicate stronger adaptation effects.

Participants of the sleep group in experiment 2 were given 90 min of sleep opportunity between adaptation and test. Polysomnography data were recorded to monitor sleep. Table 1 summarizes the average absolute and relative times spent in each sleep stage. Bivariate Pearson correlation analysis of individual adaptation effects (FMR) and sleep parameters showed that the amount of rapid eye movement (REM) sleep was predictive of an individuals' adaptation effect (*r* = 0.70; *p* = 0.025; figure 3 and table 1). Participants who exhibited a greater amount of REM sleep showed larger adaptation effects after sleep than participants who experienced no or only little REM. The reverse pattern was found for slow wave sleep (SWS; *r* = −0.89; *p* < 0.001), indicating a negative relationship of SWS duration and the size of behavioural effects. The times spent in REM correlated negatively with SWS duration (*r* = −0.70; *p* = 0.017). Greater behavioural effects were further associated with a higher spindle density of slow spindles (9–12 Hz; *r* = −0.63; *p* = 0.034) and a lower spindle density of fast spindles (12–15 Hz; *r* = 0.64; *p* = 0.038; figure 3). These *p*-values are uncorrected for multiple comparisons.

**Table 1.** Sleep data. (Total sleep time corresponds to the total time in bed minus wake epochs. Relative times represent the ratio between the total times per sleep stage and total sleep time per participant. Statistics (*r* and *p*) are based on the absolute values.)

sleep parameters	absolute time in epochs (mean±s.e.m.)	relative time in % (mean±s.e.m.)	*r*	*p*
total sleep time	159.91±4.86	100±0	0.32	n.s.
S1	35.10±4.87	20.58±2.94	0.14	n.s.
S2	60.20±9.67	36.51±4.90	0.63	n.s.
S3	12.50±2.70	8.21±1.54	0.25	n.s.
S4	31.60±11.21	20.99±6.50	−0.88	<0.001
SWS (S3 and S4 collapsed)	44.10±11.65	29.21±6.74	−0.89	<0.001
REM	11.40±4.10	7.50±2.46	0.70	0.025
spindle density (9–12 Hz)			−0.63	0.038
spindle density (12–15 Hz)			0.64	0.034

**Figure 4.**
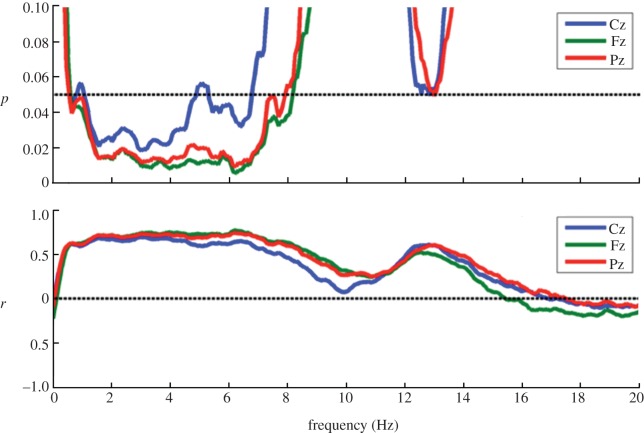
Frequency analysis. Post-sleep adaptation was associated with lower delta and theta frequency power over Fz, Cz and Pz electrodes. This is indicated by a positive correlation of fitted mean rating (FMR) and the data based on the frequency analysis. Low FMRs represent small behavioural effects, and high FMRs represent large behavioural effects. (Online version in colour.)

Full spectral analysis was performed to test for associations of sleep EEG frequency power and behaviour. Collapsed across all sleep stages, delta (less than 2 Hz) and theta (3–7 Hz) power at frontal, central and parietal electrodes (i.e. Fz, Cz, Pz) were negatively associated with the size of behavioural effects, as indicated by a positive correlation of FMRs and these parameters (figure 4).

## 4. Discussion

The results of two experiments provide evidence for a powerful association between sleep and sensory adaptation. Face distortion after-effects measured after 12 h or 90 min were larger in groups of participants who slept between adaptation and test compared with other groups of participants who stayed awake. Participants were adapted to distorted images of famous faces and negative after-effects in the perception of the original face were measured after adaptation. Highly familiar faces of celebrities were used to ensure the presence of strong and stable representations prior to adaptation.

So far, the concepts of sleep and adaptation have not been directly linked. One recent study found that the McCollough effect persisted over a period of sleep [27]. However, in this previous study [27], blindfolding participants had similar effects which makes it difficult to argue that sleep is a factor in adaptation consolidation that goes beyond merely blocking interfering visual information. On the other hand, reduced visual interference, also known as storage [3,25,26], cannot solely explain the very long-lasting adaptation effects that were found up to weeks and months after adaptation [10–12]. Most studies testing for long-term after-effects did not include experimental manipulations of visual input following adaptation. In other words, adaptation can be maintained even in the absence of experimentally induced storage. In addition, studies testing specifically for storage effects usually reported that adaptation effects decrease and return to baseline levels quickly as soon as a given storage period is finished. Thus, storage is usually seen as a mechanism that delays the onset of the natural decay process in adaptation rather than modulating (i.e. attenuating) it also in the long run. The question here is whether sleep represents something qualitatively different from low-interference waking and if it has an additional long-term purpose in the consolidation of adaptation.

Importantly, however, in the high-level adaptation experiments reported here no storage was seen during waking, even when participants were blindfolded. Therefore, the finding that sleep facilitated face adaptation cannot be explained by reduced visual interference but instead seems to lie within the neurophysiological properties of sleep.

We expected that REM sleep played an essential role in adaptation, based on previous studies suggesting an association of SWS (stages 3 and 4) and the consolidation of declarative memories, whereas REM seems to be more strongly associated with procedural information, perceptual learning and recognition memory for faces [36]. This is also what was found here. Participants who exhibited high amounts of REM sleep also showed greater adaptation. The average time spent in REM was negatively correlated with SWS duration, which, in turn, can explain the negative correlation that was found for SWS duration and adaptation and for low-frequency power in the delta and theta range and adaptation. Adaptation was further predicted by spindle density of both slow and fast spindles, however in opposite ways. A high density of slow spindles positively correlated with behaviour. At the same time, a high density of fast spindles showed a negative correlation with post-sleep adaptation effects. While the general importance of sleep spindles in memory consolidation processes has been highlighted repeatedly [37], our understanding of the differences in the functional roles of different types of sleep spindles (i.e. fast and slow spindles) is less complete. Some studies reported differential effects of fast and slow spindles and concluded that fast spindles seem to play a major role in memory consolidation, whereas the role of slow spindles is ambiguous [34,38]. Compared with these reports, our findings seem to go in the opposite direction and suggest that slow spindles are directly linked to the retention of face adaptation. Adaptation and memory consolidation are very different cognitive phenomena and it is therefore likely that these mechanisms are associated with different sleep parameters. Future research is warranted to establish the causal links between sleep parameters and adaptation consolidation.

The experiments in this study did not include measurements of individual baselines of the size of after-effects immediately after adaptation which made it difficult to precisely assess the impact of sleep on a particular individual's perception. This decision was based on previous research showing that multiple testings after adaptation can quickly reduce or abolish initial long-term effects [12]. However, experiment 2 included a between-subjects control group with no interval between adaptation and test to investigate whether the observed differences between sleep and wake groups resulted from absolute increases in adaptation after sleep compared with baseline or rather from reduced decay during sleep. The effects of the sleep group were essentially similar to those of the control group and the data of the wake group suggested a decay of adaptation, although this difference was not statistically significant. However, this observation is supported by the fact that adaptation was not stronger after approximately 8 h of sleep compared with 90 min which indicates that adaptation is not a function of sleep duration. Thus, our results are more compatible with the hypothesis that sleep prevents decay.

In conclusion, this study shows a direct association between sleep and the integration of visual adaptation. Our findings suggest a substantial role of sleep in the processing and the organization of previous sensory experiences. Given the automatic and permanent adaptive characteristics of the sensory systems, this role extends the classic sleep and memory association with lower-level sensory processes not usually considered as mechanisms associated with mnemonic processes.

## Notes

This study was approved by the University College London (UCL) ethics committee.
